# Pembrolizumab: A reliable second‐line treatment option for advanced esophageal cancer

**DOI:** 10.1111/1759-7714.14395

**Published:** 2022-03-20

**Authors:** Li Zheng, Yabi Zhu, Yangyang Liu, Xu Ma, Fang Xu

**Affiliations:** ^1^ Department of Gastroenterology Lishui People's Hospital Zhejiang China

To the editor

Esophageal cancer is the sixth leading cause of carcinoma‐associated death and the seventh most clinically diagnosed carcinoma globally, accounting for 544 076 new deaths and 604 127 new cases in year 2020.^1^ About half of the esophageal cancer cases are diagnosed in China, with 70% of the cases at advanced stages where the opportunity for surgery has been lost. Although chemoradiation can prolong overall survival and improve the locally controlled rate of esophageal cancer, the prognosis of advanced esophageal carcinoma has not significantly improved for a long time. Recently, immune checkpoint inhibitor (ICI) immunotherapy, a new cancer treatment choice, has provided a promising treatment method for a variety of malignant carcinomas and has greatly improved the survival of patients with malignant melanoma, lung cancer, cervical cancer, bladder cancer, and other carcinomas.

Several clinical trials have evaluated the treatment efficacy and safety of the ICI pembrolizumab for second‐line treatment of advanced esophageal cancer.^2^, ^3^, ^4^ In a multiple cohort, a phase IB clinical study of cases with programmed death ligand‐1 (PD‐L1)‐positive advanced esophageal cancer (KEYNOTE‐028), patients received pembrolizumab 10 mg/kg every 2 weeks up to 2 years or until confirmed disease progression or intolerable toxicity.^3^ The results indicated that the objective response rate (ORR) was 28% with a median maintained response of 15 months and median overall survival (OS) and progression‐free survival (PFS) of 7 and 1.8 months, respectively. Severe adverse events or death were not recorded in pembrolizumab group. KEYNOTE‐028 preliminarily demonstrated the efficacy and safety of pembrolizumab in second‐line treatment of advanced esophageal cancer. A phase II clinical study to evaluate the efficacy and safety of pembrolizumab for second‐line treatment of advanced esophageal cancer (KEYNOTE‐180) further confirmed the potential benefits of pembrolizumab with durable antitumor activity and manageable safety in patients with pretreated advanced esophageal cancer.^4^ More reliable evidence from a phase III clinical trial (KEYNOTE‐181) confirmed pembrolizumab prolonged OS versus chemotherapy as second‐line therapy for advanced esophageal cancer in patients of PD‐L1 combined positive score (CPS) ≥10, with fewer treatment‐related adverse events.

In our view, this series of clinical trials from phase I to phase III provide reliable evidence of the efficacy and safety of pembrolizumab as a second‐line treatment of advanced esophageal cancer cases with PD‐L1 CPS ≥10. However, survival advantages were not found for cases of PDL‐1 CPS <10, adenocarcinoma, female, and extra‐Asia cases.^2^ Therefore whether the nonsuperior results for the aforementioned cases are for pembrolizumab itself or a small sample size of subgroup analysis needs further investigation from larger sample size clinical trials.
